# Enhancing Mechanical Properties of Polymer 3D Printed Parts

**DOI:** 10.3390/polym13040562

**Published:** 2021-02-13

**Authors:** Catalin Gheorghe Amza, Aurelian Zapciu, George Constantin, Florin Baciu, Mihai Ion Vasile

**Affiliations:** Faculty of Industrial Engineering and Robotics, University “Politehnica” of Bucharest, 060042 Bucharest, Romania; aurelianzapciu@yahoo.com (A.Z.); icmas74@yahoo.de (G.C.); florin.baciu@upb.ro (F.B.); vasileionmihai@yahoo.com (M.I.V.)

**Keywords:** 3D printed, PETG, polymer remelting

## Abstract

Parts made from thermoplastic polymers fabricated through 3D printing have reduced mechanical properties compared to those fabricated through injection molding. This paper analyzes a post-processing heat treatment aimed at enhancing mechanical properties of 3D printed parts, in order to reduce the difference mentioned above and thus increase their applicability in functional applications. Polyethylene Terephthalate Glycol (PETG) polymer is used to 3D print test parts with 100% infill. After printing, samples are packed in sodium chloride powder and then heat treated at a temperature of 220 °C for 5 to 15 min. During heat treatment, the powder acts as support, preventing deformation of the parts. Results of destructive testing experiments show a significant increase in tensile and compressive strength following heat treatment. Treated parts 3D printed in vertical orientation, usually the weakest, display 143% higher tensile strength compared to a control group, surpassing the tensile strength of untreated parts printed in horizontal orientation—usually the strongest. Furthermore, compressive strength increases by 50% following heat treatment compared to control group. SEM analysis reveals improved internal structure after heat treatment. These results show that the investigated heat treatment increases mechanical characteristics of 3D printed PETG parts, without the downside of severe part deformation, thus reducing the performance gap between 3D printing and injection molding when using common polymers.

## 1. Introduction

**Figure 1:**
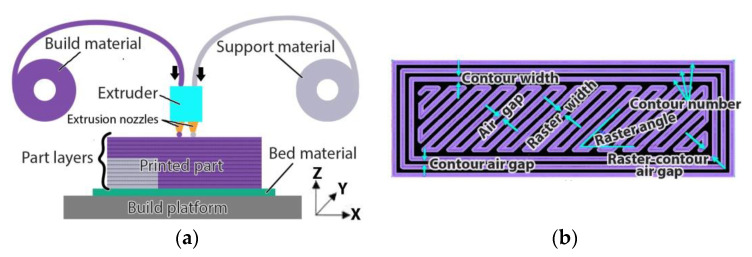
Material extrusion 3D printing: (**a**) Process schematic—the extruder and the build platform move relative to one another in the XY plane in order to deposit a horizontal layer and in the Z direction in order to position for the next layer; (**b**) Process parameters that influence horizontal layer deposition.

The era of rapid prototyping started in the 1980s with the emergence of stereolithography, a process that used a laser to selectively cure photosensitive polymer [1]. A decade later, in 1992, the American company Stratasys introduced a new manufacturing process called Fused Deposition Modeling (FDM) [2]. This new process used thermoplastic filament that is heated up to near its melting point and then extruded and deposited on a surface, forming horizontal layers of material. In the following years, other processes, materials and capabilities were developed and new use cases other than rapid prototyping became possible. Thus, the industry began to adopt the term “additive manufacturing” (AM) as a more encompassing name than “rapid prototyping”. In 2009, patents held by Stratasys on FDM expired, enabling others to bring alternatives to the market, leading to widespread adoption of this manufacturing process. Hobby-level users and small companies alike started developing and commercializing 3D printing machines that use filament extrusion. As the name “FDM” is still a Stratasys trademark, the process is now recognized as material extrusion 3D printing (ME3DP). Thanks to its relative ease of use, ease of sourcing build materials and low cost of hardware equipment, ME3DP is now extensively used by home users and businesses of all sizes [3,4,5] Manufacturing of parts using this process is done by depositing horizontal layers of material, one on top of another, each layer being made of extruded thermoplastic feedstock. The feedstock usually comes in the form of a thin filament when used for fabricating small and medium-sized parts or in the form of pellets, when used to produce larger parts. The feedstock is first heated up to a temperature that is usually between the glass transition temperature of the material and its melting point and then it is deposited following a path controlled by a computer. The first horizontal layer of material is deposited onto a platform or bed that moves in the horizontal plane relative to the extruder. After depositing the first layer, the distance between the extruder and the platform is increased by an amount equal to the layer height and a new layer of material starts being deposited. A schematic of a 3D printer that works based on the ME3DP principle is shown in Figure 1a. The extrusion path (the motion of the extruder relative to the platform) is calculated by a computer based on multiple process parameters including nozzle and feedstock filament sizes, fill pattern and orientation, number of exterior contours and their width, etc. Some of the key process parameters that determine how individual horizontal layers will be deposited are shown in Figure 1b.

While 3D printing processes allow for a broad range of geometries to be fabricated, the mechanical properties of 3D printed parts made from polymers are usually inferior to those of parts fabricated through injection molding [6,7]. This leads to a diminution in the advantages brought by the complex geometry of parts, such as lower mass. It is very well documented that 3D printed parts made through ME3DP (or FDM) have anisotropic mechanical properties [8,9] meaning that parts may have a preferable orientation during fabrication depending on their intended purpose. If maximum strength is desired in more than a single plane, 3D printing might produce parts that are insufficiently strong due to the anisotropic characteristics introduced by the fabrication process. This limits the potential of fabricating functional 3D printed parts for applications such as brackets, supports, mounting jigs, and many others.

The weak mechanical properties of 3D printed parts compared to injection molded parts are caused by inadequate adhesion between deposited filaments and between stacked layers. ME3DP relies on heating material and extruding it through a small diameter nozzle. After exiting the nozzle, the material immediately starts to cool down. This produces a large difference of temperature between freshly deposited filaments and the previously deposited part structure. For that reason, 3D printed parts have lower tensile strength in the Z direction than in the X or Y directions [10]. 

One area of research is designing special formulations of thermoplastic materials specifically intended for use in 3D printing [11]. The focus is both on materials that can withstand higher temperatures, such as glass-filled polymers [12], and on new formulations of engineering plastics with better flowability such as polyether ether ketones (PEEK) [13]. A key development was the creation of materials that increase mechanical properties of a polymer matrix through the inclusion of various types of carbon fibers [14]. These novel materials seek to eliminate or reduce some of the temperature-generated problems. While successfully offering better mechanical properties, this approach comes with the downside of increased cost. This is due either to the lack of large-scale production of the novel materials or to the increased capability requirements of 3D printers, in the case of materials with higher extrusion temperatures [15]. Additionally, creating new types of materials adds to the issues of recycling plastic waste [16], as most often materials with superior mechanical and chemical characteristics are not readily recyclable or biodegradable [17]. Work done by Tian et al. investigated the recycling of polylactic acid (PLA) composites with continuous carbon fiber reinforcement and the mechanical properties of re-manufactured parts [18], achieving a material recovery rate of 100% for continuous carbon fiber and 73% for PLA matrix. Zhao et al. studied the ability of 3D printed parts made from PLA material to be recycled in a closed-loop process. Their study found that the material degrades during the recycling process and that virgin material needs to be added in order to preserve printability and good properties [19].

According to research conducted by Dawoud et al. on mechanical properties of ABS [20], 3D printed parts exhibit more internal voids compared to injection molded parts due to the lack of externally applied pressure during fabrication. While this effect can be reduced by lowering the layer height, the resulting micro-voids still have an important effect on part strength. This effect is enhanced by the repeating aspect of 3D printing that favors the formation of aligned micro-voids patterns [21]. Variation of the infill patterns can help redistribute the micro-voids. Researchers have investigated heat-treating 3D printed parts as a method of increasing interlayer adhesion and of reducing the internal thermal stresses resulted during manufacturing [22]. While annealing 3D printed parts does produce parts with improved mechanical properties, having higher strength and stiffness [23], it often introduces a new problem, which is the deformation of the part during heat treatment due to realigning of the polymer’s molecular chains and the orthotropic character of the part [24]. This deformation is hard to predict and mitigate before the 3D fabrication process because of the anisotropic behavior mentioned previously [25].

Poor interlayer tensile strength resulting from reduced adhesion generated by temperature differences between layers when depositing material is specific to ME3DP fabrication. As filaments of molten material are being deposited, they begin cooling, creating a temperature difference relative to that of newly extruded material. The research of Morales et al. shows that the increase of the temperature difference between successive layers negatively influences the mechanical properties of the printed part [26].

Polyethylene Terephthalate Glycol (PETG) is a naturally transparent, glycol-modified form of polyethylene terephthalate (PET), the most widely produced polymer in the world. This thermoformable thermoplastic shares many of the properties of PET [27,28] and is widely used in a broad range of applications [29,30,31]. Due to its mechanical and thermal properties, it is used in 3D printing, gaining a sizable market and becoming the third most used polymer in this field, behind ABS (acetyl-butadiene-styrene) and PLA.

Work by Bhundari et al. describes the behavior of PETG and PLA during annealing [32]. PETG is an amorphous polymer with very low crystallization levels and the reduction in interlayer tensile strength characteristic to ME3DP fabrication is recovered by post-processing when the annealing temperature is higher than the glass transition temperature. In the case of PLA, which is a semi-crystalline polymer, the recovery of the interlayer tensile strength is observed when the annealing temperature is between the glass transition temperature and the cold-crystallization temperature.

This study looks to assess whether embedding 3D printed parts in a powder bed, followed by heat treatment will result in superior mechanical properties such as tensile and compressive strength. The bed full of powder in this process serves a double purpose, first to constrain the part and prevent deformation, and second, to distribute heat evenly and prevent the accumulation of thermal stresses inside the treated part.

## 2. Materials and Methods

The material used for fabricating test parts and test samples for this study is transparent PETG filament, undyed, 1.75 mm in diameter, manufactured by Prima Printer Nordic AB (Malmö, Sweden) and commercialized under the brand name “PrimaSelect”. The material has a glass transition temperature of 86 °C and a melting temperature of 255 °C. The slicer software used to split the 3D models into horizontal layers is Cura 4.5 by Ultimaker (Geldermalsen, The Netherlands). Samples were 3D printed on a Creality Ender 3 3D printer (Shenzen, China) equipped with a 0.4 mm diameter nozzle.

**Figure 2:**
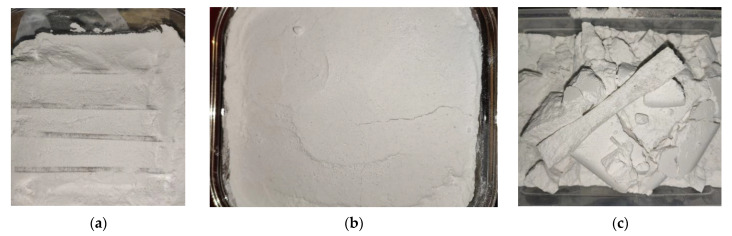
Heat treatment: (**a**) Samples being powder-packed in the borosilicate glass recipient; (**b**) Recipient with packed powder; (**c**) 3D printed samples after heat treatment.

After 3D printing of sample part sets with 100% infill, a heat treatment process was performed. The printed parts were placed on a powder bed inside a borosilicate glass container and then they were covered with more powder. The powder used in the experiment is grounded table salt, composed of NaCl, KIO_3_ 42–67 mg/kg, E536 anti-caking agent, supplied by Salina Ocna Dej (Dej, Romania). This is an inexpensive material, easy to grind into small particles, non-toxic and resistant to the process temperatures. After grinding, the salt powder particles were separated using a mesh 270 sieve, resulting in particles 53 µm or smaller. The resulting powder was kept at 200 °C for 30 min to eliminate moisture, then it was cooled down in a sealed recipient. An alternative material for the powder is Plaster of Paris (calcium sulfate hemihydrate). A rubber mallet was used to densely pack the powder and attention was paid when filling holes and around the edges of the parts (Figure 2). Thermistors used to monitor the powder temperature were placed in the powder bed and packed together with the samples.

The packed samples were introduced into a convection oven set at 220 °C, a temperature that is significantly above the glass transition temperature of the material but below its melting temperature. A small metal plate weighing 1.5 kg was placed on top of the powder in order to provide compression. Once the temperature inside the powder reached 220 °C, a timer was started. Two treatment lengths were trialed, with the parts held at 220 °C for 5 min (partial treatment) or for 15 min (full treatment). In each case, the borosilicate glass container was removed from the oven at the end of the treatment and left to cool at room temperature (24 °C) before unpacking (Figure 2c).

**Figure 3:**
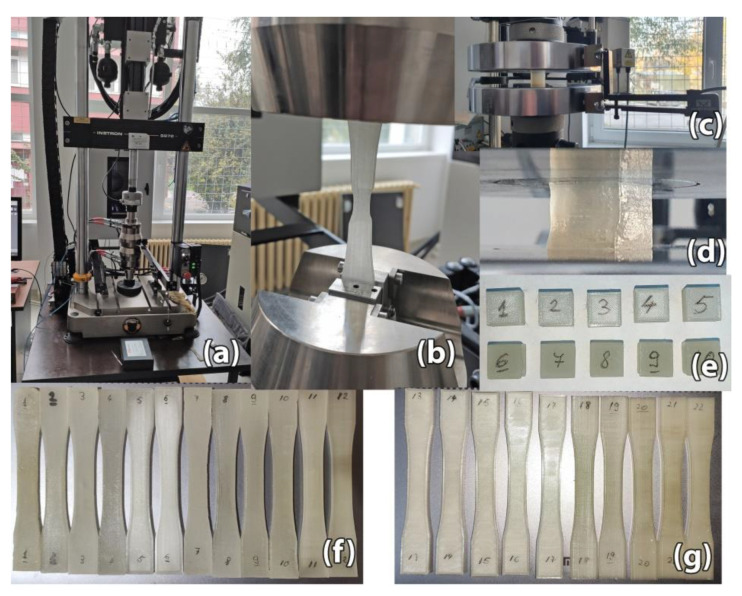
Destructive tests: (**a**) Instron 8872 machine; (**b**) Tensile strength testing; (**c**) Instron 8801 universal testing machine; (**d**) Compressive strength testing; (**e**) Cubic samples for compressive strength testing. Samples #1–#5 belong in the control group while samples #6–#10 are in the heat treatment group; (**f**) Heat treated samples for tensile strength testing. Samples #1–#4 and #7–#10 have undergone full heat treatment while samples #5–#6 and #11–#12 went through partial heat treatment. Samples #1–#6 are printed in horizontal orientation while #7–#12 are printed in vertical orientation; (**g**) Control group for tensile strength testing. Samples #13–#17 are printed in horizontal orientation while #18–#22 are printed in vertical orientation.

A set of destructive tests were performed on 3D printed samples to determine the post-processing heat treatment effect on tensile and compressive strength. Tensile strength testing was performed according to ASTM D638-14 [33] on 22 samples of Type I standardized dimensions, with a section size of 13 mm × 4 mm (Figure 3f,g). For compressive strength testing, 10 cube samples were fabricated, with dimensions of 10 × 10 × 10 mm^3^ (Figure 3e). The cube samples were maintained at 220 °C for 15 min. All sample parts were 3D printed with 100% infill alternating between 45° and −45° raster angle.

An important aspect of ME3DP is that results are dependent on the machine used for 3D printing, as revealed by a review of Popescu et al. [34]. This factor is worth considering, as the process described in this paper requires the production of parts with 100% infill and as few internal voids as possible. Using the implicit values for slicing parameters that influence part infill might not be sufficient, thus, more accurate control is necessary. Parameters such as material flow percentage and infill overlap with the exterior contours need to be adjusted individually for each 3D printer. In this study, material flow percentage, a parameter that changes the volume of extruded material, was set at 103%, while infill overlap, which sets the overlap distance between filaments that form the exterior contour and filaments that form the infill, was set at 25%.

The main parameters used for the 3D printing process are shown in Table 1.

The 3D printed specimens were tensile tested on an Instron 8872 (Norwood, MA, USA) universal testing machine, at a speed of 10 mm/min and a preload force of 5 N (Figure 3a,b). Compressive strength testing of the 10 cube samples was done on an Instron 8801 universal testing machine (Figure 3c,d). During compressive strength testing the compressive load was applied along the *Z*-axis of the fabricated cube samples.

The results of tensile strength and stiffness testing are presented in Section 3.1, while the results of compressive strength testing are discussed in Section 3.2.

In order to investigate the changes occurring on the external surfaces and within the internal structure of the treated parts, a Scanning Electron Microscopy analysis (SEM) was performed. For this analysis, one sample was selected and investigated from each group (fully treated, partially treated, control). The morphology of the samples was analyzed using a Quanta Inspect F50 FEG (Field Emission Gun) scanning electron microscope with 1.2 nm resolution, equipped with an energy-dispersive X-ray (EDX) analyzer (resolution of 133 eV at MnKα, Thermo Fisher Scientific, Eindhoven, The Netherlands). All samples were covered with a slim layer of Au using the Q150 PlusSeries from Quorum Technologies (Lewes, UK). The sample coverage time was 30 s. The results are discussed in Section 3.3.

In addition to the tests performed on standardized samples, more 3D printed parts were manufactured to assess the capability of the support powder to prevent deformation during heat treatment. Several geometries were tested, including supported arches, thin structures, curved and flat surfaces.

## 3. Results

### 3.1. Tensile Strength of Thermally Treated Parts Made from PETG

**Figure 4:**
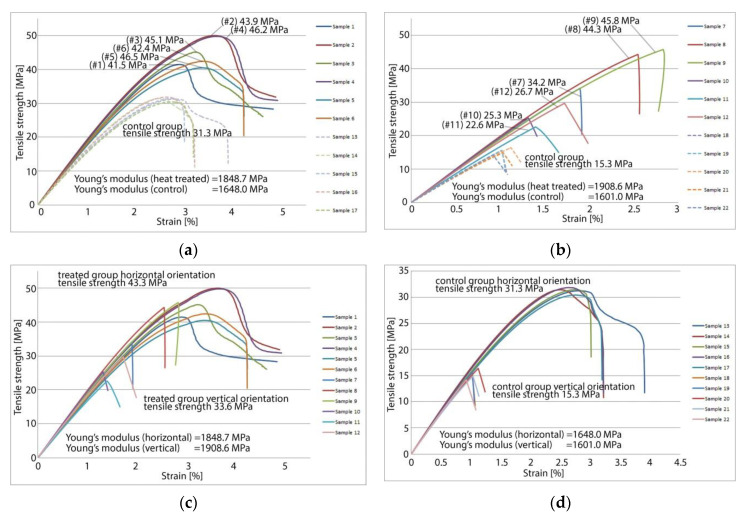
Tensile strength testing results: (**a**) Samples printed horizontally treated vs. control group; (**b**) Samples printed vertically treated vs. control group; (**c**) Treated samples printed vertically vs. horizontally; (**d**) Control group samples, printed with the same set of parameters but not heat treated, showing the poorest mechanical characteristics.

For the 12 sample specimens manufactured for tensile testing no significant deformation was noticed following heat treatment. A common mode of failure when annealing long and thin parts is that of bending. This was also absent after heat treating the samples using the method described in this paper. The results of tensile strength testing are shown in Figure 4. Destructive tensile testing reveals that heat treated 3D printed PETG samples have a significant increase in strength and in stiffness compared to an untreated control group of samples fabricated on the same 3D printer with an identical set of parameters.

**Figure 5:**
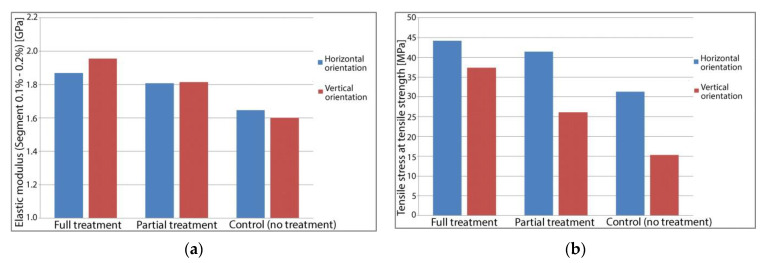
Tensile strength testing results—process averages: (**a**) Average Young’s Modulus for tested parts; (**b**) Average tensile strength for tested parts.

Tensile testing results averaged for each sample group are presented in Figure 5. Heat treated parts show significantly higher tensile strength, with fully treated parts exhibiting 41.1% increased strength over the control group in the case of horizontal orientation and 143.9% increased strength over the control group in the case of samples printed in vertical orientation. In addition to being stronger, the treated samples are also stiffer. Young’s modulus of elasticity is 13.3% higher for fully treated horizontal orientation samples and 22.1% higher for fully treated vertical orientation samples. There is a correlation between the length of the heat treatment and the observed mechanical properties. For fully treated samples printed horizontally, Young’s Modulus is on average 3.5% higher than for partially treated samples, this difference increasing to 7.4% for parts printed in a vertical orientation. 

Similar outcomes were found for tensile strength, with fully treated horizontal orientation samples being 6.5% stronger than their partially treated correspondents. The difference increases to 42.9% for vertically printed samples. The average values of the determined mechanical properties are shown in Table 2.

### 3.2. Compressive Strength of Thermally Treated Parts Made from PETG

**Figure 6:**
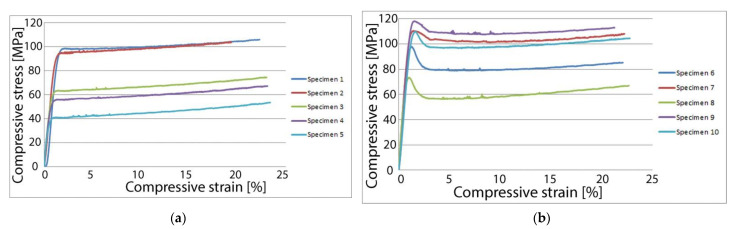
Compressive strength testing results: (**a**) Compressive strength of the 5 samples from the control group varied between 41.3 MPa and 98.4 MPa; (**b**) Compressive strength of the 5 samples from the heat-treated group varied between 73.4 MPa and 118.1 MPa.

Destructive compressive testing shows a significant increase in compressive strength, with heat treated samples achieving up to 118 MPa of compressive stress before failing. Samples from this group achieve on average 43.7% more compressive stress before failing compared to the control group. The mode of failure also differs between the two sample groups, with the heat-treated samples experiencing some shearing under compressive load, while the control group exhibits a more linear plastic deformation. This further validates the results found following the tensile tests that showed a slight increase in tensile stiffness for the heat-treated parts. The results of compressive strength testing for individual samples are shown in Figure 6. 

**Figure 7:**
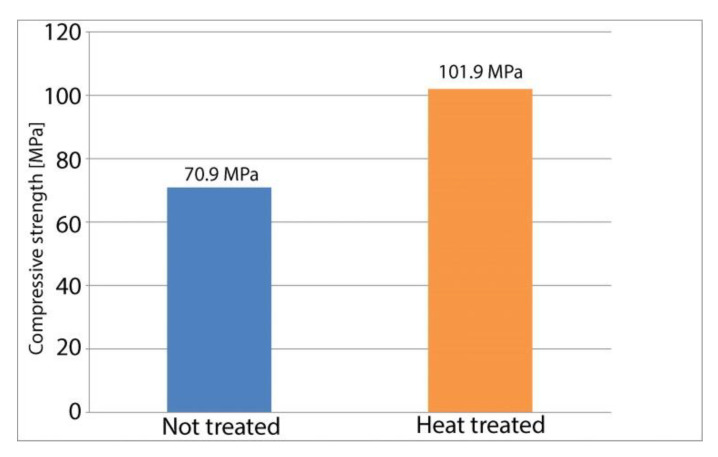
Compressive strength—sample averages.

Figure 7 depicts a column chart for average values found during compressive testing of the heat-treated samples and of the control group. Table 3 also includes the standard error of the means.

### 3.3. Scanning Electron Microscopy Analysis

**Figure 8:**
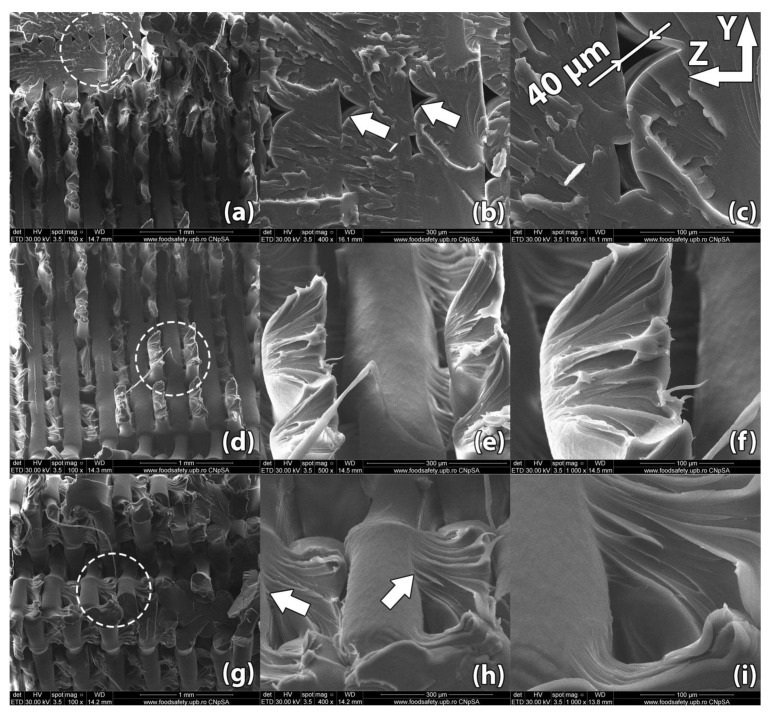
Scanning Electron Microscopy image of a control sample printed in horizontal orientation (sample #14): (**a**–**c**) Due to the raster angle changing from 45° to −45° for each horizontal layer, internal voids 20–50 µm in width remain at the interface of the deposited filaments; (**d**–**f**) Upon rupture, the sample presents fracture lines along the deposited layers in a zig-zag pattern specific to the −45°/45° infill used in part manufacturing; (**g**–**i**) Interlayer fusion is limited to the areas where the deposited filaments of two horizontal layers intersect.

**Figure 9:**
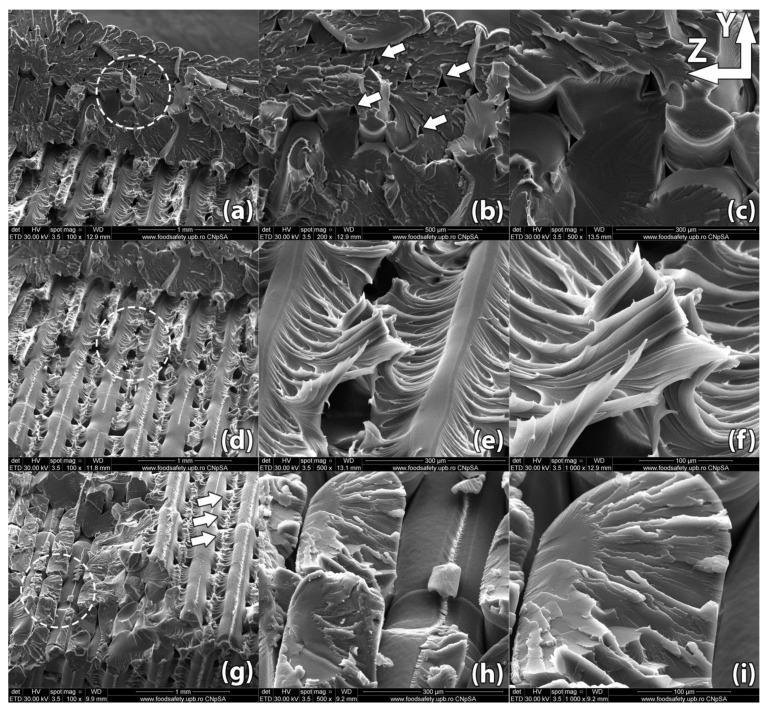
Scanning Electron Microscopy image of a sample printed in horizontal orientation (sample #05). (**a**–**c**) Due to the raster angle changing from 45° to −45° for each horizontal layer, internal voids remain at the interface of the deposited filaments but their size is significantly reduced compared to the control sample, ranging between 15 µm and 30 µm in width; (**d**–**f**) Upon rupture, the sample presented fracture lines along the deposited layers in a zig-zag pattern specific to the −45°/45° infill used in part manufacturing; (**g**–**i**). Interlayer fusion is increased compared to the control sample.

**Figure 10:**
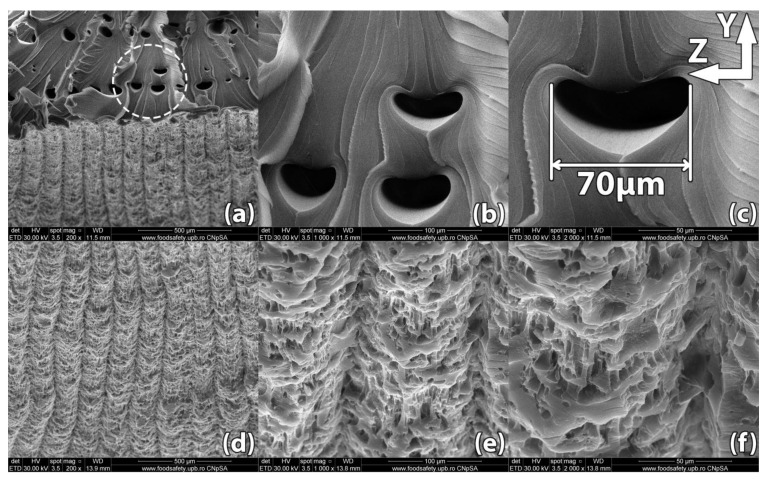
Scanning Electron Microscopy image of a sample printed in horizontal orientation (sample #03): (**a**–**c**) Merged internal voids of an obloid shape, 50–75 µm in length, form at the interface between raster angles changes; (**d**–**f**) The exterior surface of the part shows micro-voids formed during the heat treatment at the interface between the sample wall and the supporting powder. These voids are 5–10 µm in size.

**Figure 11:**
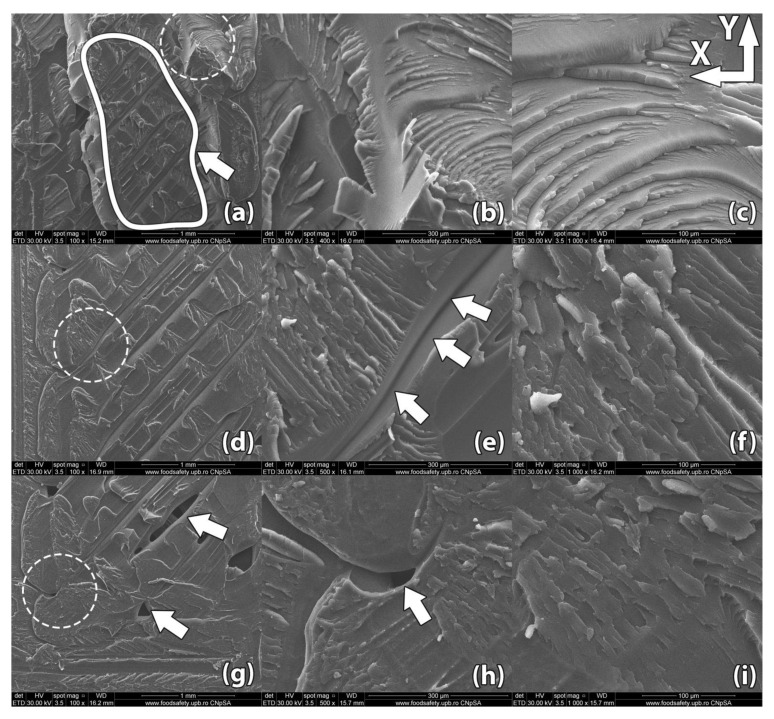
Scanning Electron Microscopy image of a control sample printed in vertical orientation (sample #18): (**a**–*c*) Upon rupture, the sample presented uniform fracture lines along the deposited horizontal layers, showing poor adhesion at the layer level; (**d**–**f**) Internal gaps between deposited filaments remained embedded in the part, weakening the structure; (**g**–**i**) Bigger internal voids remained embedded in the part in the area where the raster angle changes from one layer to another.

**Figure 12:**
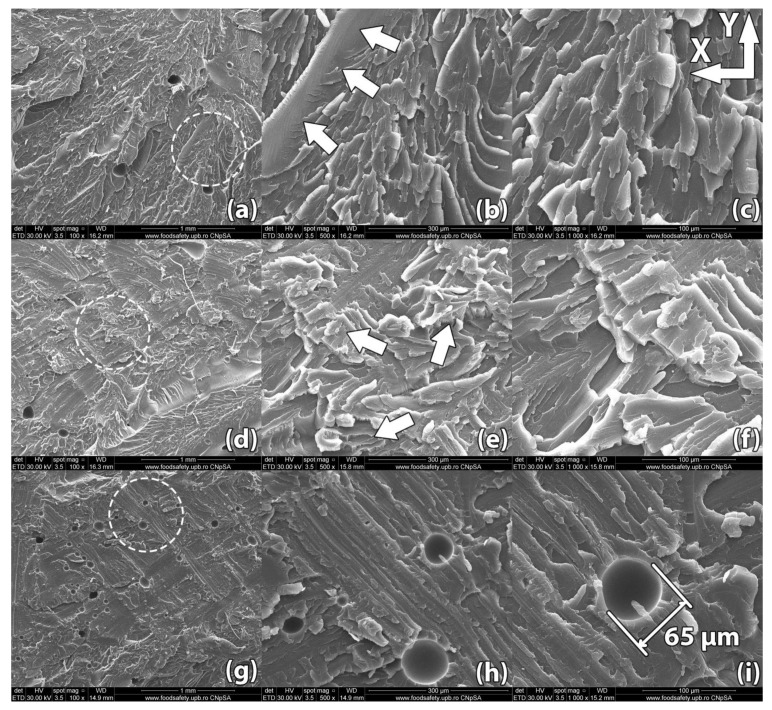
Scanning Electron Microscopy image of a sample printed in vertical orientation (sample #09): (**a**–**c**) Unlike the control sample, the linear voids between deposited filaments have been filled during the heat treatment; (**d**–**f**) Upon rupture, the sample presented fracture lines along multiple horizontal layers, indicating superior layer adhesion; (**g**–**i**) Spherical pockets of air 10–70 µm in diameter remain inside the part.

Images of the samples’ fracture surfaces taken through scanning electron microscopy reveal a series of mechanisms that occurred during heat treatment and led to the results found through destructive tests. Figure 8, Figure 9, Figure 10, Figure 11 and Figure 12 show images of various surfaces of the investigated samples taken at 3 different resolutions corresponding to magnification levels of 100×, 500× and 1000×. The acceleration level has been set at 20 kV.

In ME3DP, macroscopic structural factors resulting from the material deposition path influence the mechanical properties of a fabricated sample just as much as the inherent properties of the material. Examples of such structural factors are the development of micro-voids between adjacent filaments, preferential arrangement of these voids along the directions of filaments, and level of diffusion at adjacent filament interfaces.

Figure 8 shows the scanning electron microscopy of sample #14 from the control group, 3D printed in a horizontal orientation. The sample ruptured in a zigzag pattern along the 45° direction determined by the horizontal layer (XY plane) infill raster angle. The criss-crossing of deposited filaments in incremental horizontal layers is what gives parts fabricated in this orientation their increased strength. This comes, however, with the disadvantage of internal void alignment along the filament directions. This effect, combined with reduced contact surface between layers resulting from the 45° alternating infill pattern, weakens the part. 

SEM analysis reveals incomplete layer adhesion and internal voids with widths ranging from 20 to 50 µm remaining due to the shifting raster angle of each successive layer. During fabrication, semi-melted filament is extruded from the nozzle. Through the interaction (convection) with the environment (printing chamber) and with the previously deposited material, a radial temperature gradient is produced as the filament cools down and solidifies. The outer surface of the filament cools down faster, as convection with the surrounding air is the dominant phenomenon, while the midsection of the filament cools down mainly through heat conduction with the surrounding filaments [35,36]. The non-uniform temperature across the filament results in non-uniform mechanical properties. The core of the filament develops stronger bonds among molecular chains. This non-uniformity produces better plastic deformation of the inner filament leading to local necking of the sample under tensile stress (Figure 8e). Small areas, where material diffuses into adjacent filaments, appear near the ends of the fractured filaments (Figure 8h).

Figure 9 shows the scanning electron microscopy of sample #5, 3D printed in a horizontal orientation and then heat treated for 5 min at 220 °C.

The sample ruptured in a zigzag pattern along the 45° direction determined by the horizontal layer infill raster angle. SEM analysis reveals that interlayer adhesion improved significantly over the control sample. The non-uniformity introduced by the temperature gradient described previously is significantly reduced after heat treatment. As a consequence, virtually no localized necking is present in the treated part and the ends of the fractured filaments begin showing signs of flaking (Figure 9i). When the heat treatment temperature passes the glass transition temperature of the material, the molecular motion becomes sufficient for the coalescence of voids and the diffusion of filament interfaces. Thus, the heat-treated sample displays significantly longer streaks of adhered material between the deposited filaments (Figure 9e), while the width of filaments remains constant. The width of internal voids ranges between 15 and 30 µm and is noticeably reduced compared to the size of the voids found in the untreated sample. It is therefore assumed that the structural defects of the 3D printed PETG samples can be suppressed effectively through heat treatment.

Figure 10 shows the scanning electron microscopy image of sample #3, 3D printed in a horizontal orientation and then heat treated at 220 °C for 15 min. The outer surface of the sample exhibits micro-voids of 5–10 µm in diameter formed at the contact interface with the support powder, an effect that depends on the size of the particles that form the support powder.

The internal voids typical of ME3DP appear to have shifted position and changed their shape to a more oblong one. This effect is due to increased molecular motion once the material passes its glass transition temperature. Typically, the internal voids line up forming weak points and reducing part strength. From this point of view, the changes observed in the position and shape of the voids helped improve part strength. Comparing these results with those found in Figure 8 and Figure 9 depicting samples #14 and #5, it can be easily observed that increasing the heat treatment time from 5 min to 15 min produced a significant effect on part internal structure. 

Figure 11 shows an image of a control sample printed in vertical orientation (sample #18). Poor layer adhesion is very noticeable, with subsequent layers of material adhering to the previous layer only in areas where the 45° raster filaments intersect. Filament bond starts with the filament interface heating above glass transition temperature, enabling polymer flow and molecular mobility. Thermal healing or diffusion occurs at the adjacent filament interface due to close physical contact. The process ends with interface cooling below the glass transition temperature. 

During ME3DP, the semi-melted polymer is extruded at a temperature above its glass transition temperature onto lower temperature polymer surfaces. This causes the filament interface to heat up briefly and then fall below glass transition temperature, resulting in poor interlayer adhesion and strength. Long internal voids remain embedded in the part forming failure points. Bigger internal voids can be found at the transition of the infill raster from one layer to the next. These voids can be larger than 100 µm. The porosity occurs because the fabrication process involves filling a horizontal layer by extruding material through a round nozzle. Porosity is thus an inherent defect of ME3DP [37]. 

Figure 12 shows the image of a sample (#9) printed in vertical orientation and heat treated at 220 °C for 15 min. 

After rupture, the sample presented fracture lines along multiple horizontal layers, indicating superior layer adhesion compared to the previously imaged control sample. This effect happens because the filament interface temperature is raised above glass transition temperature. Unlike the thermal profile of the filament interfaces during fabrication described for sample #18, in the case of heat-treated parts the interface temperature no longer drops rapidly below glass transition temperature, allowing for more complete interface bonding [38]. In addition to the increased interlayer adhesion, void migration and coalescence due to increased molecular mobility at high temperatures can be easily observed. The long voids identified at the rupture surface of the control sample cannot be observed at the rupture interface of the heat-treated sample. Voids with a spherical shape and dimensions ranging from 10 µm to 70 µm still remain embedded in the material. These voids appear to have less of a preferred alignment compared to those found in untreated samples. These observations validate the large improvement in mechanical properties of sample #9 over the imaged sample #18, the tensile strength increasing in this case by nearly 200% (45.78 MPa vs. 15.52 MPa), while Young’s Modulus increased by 19.5% (1921.4 MPa vs. 1610.2 MPa).

## 4. Discussion

**Figure 13:**
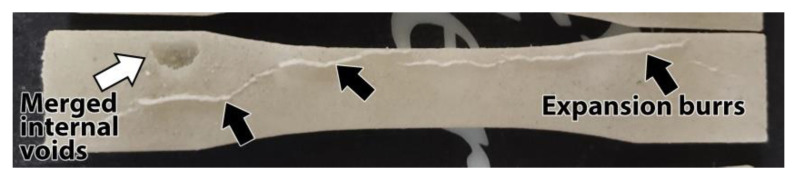
Defects appearing in heat treated part. Black arrows point to expansion burrs that occurred due to inadequate packing of powder support material. The white arrow highlights a large internal void.

Following heat treatment, several samples have developed defects resulting from improper powder packing. One such defect, shown in Figure 13, is a burr where polymer has infiltrated a narrow gap formed in the support powder. As the material expanded into this gap, a large void was also formed in another location of the part. 

This type of defect did not appear in parts that were heat treated in properly packed powder, but it underlined the importance of further refining the process and finding methods that ensure a high degree of success.

For this study a number of 22 samples with dimensions according to ASTM D638-14 Type I and 10 cubic samples with dimensions of 10 × 10 × 10 mm^3^ were 3D printed from PETG material. The samples were split into groups and 12 Type I samples and 5 cubic samples were heat treated, then tested to assess their mechanical properties relative to the control group. Results show that the heat treatment leads to samples having significantly better mechanical properties. Fully treated samples printed horizontally showed a 41% increase in tensile strength compared to the control group (44.2 MPa compared to 31.3 MPa) while an even bigger effect was observed in fully treated samples printed vertically, which showed a 143% increase compared to the control group (37.4 MPa compared to 15.3 MPa). With this large increase in tensile strength, the treated vertically printed samples surpassed the results of untreated samples 3D printed in a horizontal orientation (31.3 MPa). Thus, this inexpensive process of heating 3D printed parts embedded in packed powder virtually eliminates the downside of the anisotropic character of ME3DP manufactured parts.

In addition to the destructive tests, Scanning Electron Microscopy was performed on the samples in order to image the changes occurred during heat treatment. Following treatment, the exterior surface of the samples showed a sensible increase in roughness. This increase was validated by SEM analysis, which revealed the formation of micro-voids of 5–10 µm in size. 

According to work published by Amza et al. on 3D printed composites [39], preferred alignment of internal voids is one of the main factors that favor part failure under mechanical load. SEM analysis done in our study reveals that void shape and size change during heat treatment, reducing the prevalence of aligned weak points in parts. 

In order to demonstrate the process applicability to parts with more complex geometries, several 3D parts have been also manufactured.

**Figure 14:**
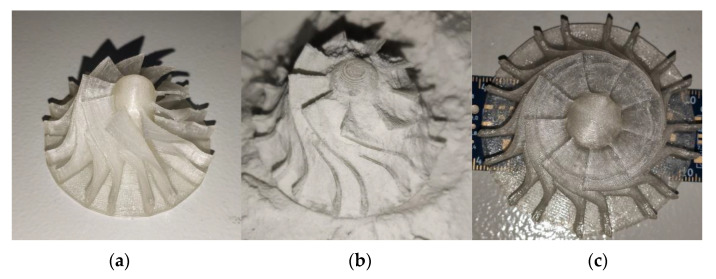
Images of a 3D printed small turbine wheel: before treatment; (**a**) and after heat treatment (**b**,**c**).

Figure 14 shows a small turbine wheel with a base diameter of 75 mm, a 2 mm thick bottom surface and with blades having width of 1.2 mm that were subjected to the process described in this paper. The part was treated for 15 min at 220 °C. Following heat treatment, the package containing the turbine wheel was left to cool at ambient temperature. After cooling, the part was unpacked, washed of any powder residue and then visually inspected. The blades and bottom of the part do not exhibit any noticeable deformation.

**Figure 15:**
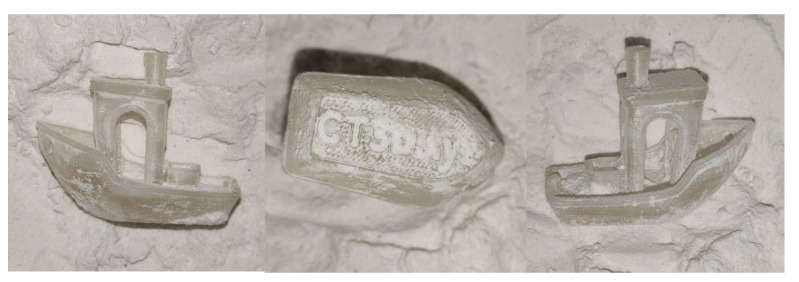
Images of a 3DBenchy (all instances shown are after heat treatment).

Figure 15 shows a heat-treated model of 3DBenchy [40], a 3D model commonly used in the 3D printing community to tune and benchmark 3D printers. The model has interior arches and complex surfaces. A similar process to that described previously has been followed. No significant deformation was observed after heat treatment. 

**Figure 16:**
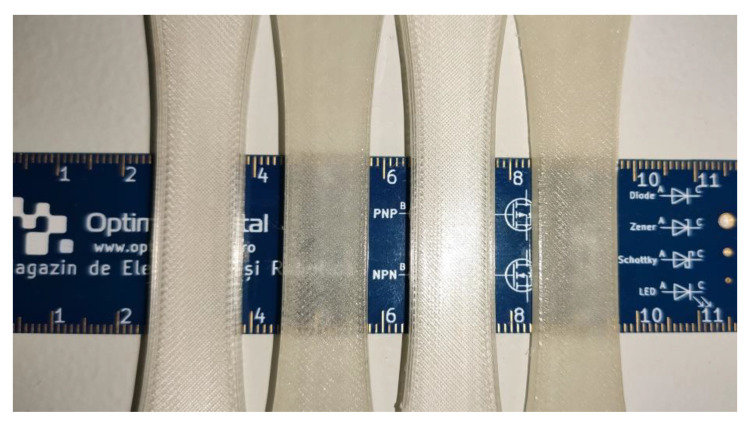
Visual changes observed in optical properties after heat treatment of horizontal orientation 3D printed parts. Lighter parts are untreated samples from the control group and darker parts are samples after full heat treatment. No other processing was done to the samples.

A consequence of the improved adhesion of the deposited filaments and filament layers is that optical properties of the sample changed after heat treatment (Figure 16), changing the parts from a translucent white to a darker, but more transparent color. This change has been observed in all treated samples, including the two 3D models shown previously. This effect could open the door to new research in the field of 3D printed lenses and other optical elements.

Other studies have pointed out that heat treatment of polymer parts also has an effect on heat resistance, not just on mechanical properties [41]. In this paper, heat treated samples made from PETG were analyzed from the viewpoint of their mechanical properties, namely tension and compressive strength. Future work could also focus on the changes in the material’s thermal behavior following post-processing.

## 5. Conclusions

This research aimed at investigating whether the mechanical properties of parts 3D printed from polyethylene terephthalate glycol-modified (PETG) polymer can be enhanced through a post-processing heat treatment. This is of interest due to the good mechanical properties needed for functional applications. 

3D printed PETG parts packed in sodium chloride powder and heat treated at 220 °C for 5 to 15 min have significantly better tensile strength and compressive strength compared to untreated parts. The results of tensile strength testing are better for samples printed in a vertical orientation, usually the weakest. After treatment, these samples showed higher strength than untreated samples printed in a horizontal orientation. This is important, as the treatment reduces the anisotropic characteristics of parts made through ME3DP. This has a positive effect on complex geometries. The length of the heat treatment correlates with better results.

SEM imaging revealed less localized necking and higher material diffusion in heat treated parts, further validating the increase in sample strength found through destructive testing. This is compatible with findings of Gao et al. who have investigated the interlayer bonds in 3D printed parts [42].

After researching heat treatment effects on dogbone-shape samples, several parts with complex geometries were 3D printed and treated in order to validate the applicability of the process to other geometries.

It can be concluded that this experimental process can be used to enhance mechanical properties of PETG. The process of packing parts in sodium chloride powder for heat treatment is inexpensive and easy to execute. However, since it involves manually placing and packing parts it is susceptible to user error. Further experimentation is desirable, investigating other polymers, packing materials and heat treatment profiles. Changes in optical properties of parts might also be of interest.

## Figures and Tables

**Table 1:** 3D printing process parameters.

Test Type	Dimensions [mm]	Extrusion Temperature [°C]	Layer Height [mm]	Infill	Exterior Contours
Tensile	13 × 4	235	0.2	100%, 45° raster	3
Compressive	10 × 10 × 10				

**Table 2:** Tensile strength and tensile stiffness of tested samples.

Tested Property	Horizontal Orientation			Vertical Orientation		
	Full Treatment	Partial Treatment	Control	Full Treatment	Partial Treatment	Control
Strength [MPa]	44.2 ± 1.0	41.5 ± 1.0	31.3 ± 0.2	37.4 ± 4.8	26.2 ± 3.5	15.3 ± 0.4
Modulus [MPa]	1868.7 ± 45.1	1808.5 ± 30.3	1648.0 ± 21.5	1955.4 ± 18.5	1815.2 ± 82.2	1601.0 ± 10.0

**Table 3:** Compressive strength of tested samples.

Property	Heat Treated	Control
Compressive strength [MPa]	101.9 ± 11.2	70.9 ± 7.8

## Data Availability

The data that support the findings of this study are available from the corresponding author upon reasonable request.

## References

[1] Hull C.W. (1984). Apparatus for Production of Three-Dimensional Objects by Stereolithography. U.S. Patent.

[2] Crump S.S. (1989). Apparatus and Method for Creating Three-Dimensional Object. U.S. Patent.

[3] Steenhuis H.-J., Pretorius L. (2016). Consumer additive manufacturing or 3D printing adoption: An exploratory study. J. Manuf. Technol. Manag..

[4] Sepasgozar S.M.E., Shi A., Yang L., Shirowzhan S., Edwards D.J. (2020). Additive Manufacturing Applications for Industry 4.0: A Systematic Critical Review. Buildings.

[5] MarketsandMarkets (2020). Market. Research Report: Industrial 3D Printing Market by Offering, Application, Process, Technology, Industry and Geography—Global Forecast to 2025.

[6] Behalek L., Safka J., Seidl M., Habr J., Bobek J. (2018). Fused Deposition Modelling vs. Injection Moulding: Influence of Fiber Orientation and Layer Thickness on the Mechanical Properties. MM Sci. J..

[7] Priya M.S., Naresh K., Jayaganthan R., Velmurugan R. (2019). A comparative study between in-house 3D printed and injection molded ABS and PLA polymers for low-frequency applications. Mater. Res. Express.

[8] Riddick J.C., Haile M.A., Von Wahlde R., Cole D.P., Bamiduro O., Johnson T.E. (2016). Fractographic analysis of tensile failure of acrylonitrile-butadiene-styrene fabricated by fused deposition modeling. Addit. Manuf..

[9] Zou R., Xia Y., Liu S., Hu P., Hou W., Hu Q., Shan C. (2016). Isotropic and anisotropic elasticity and yielding of 3D printed material. Compos. Part B Eng..

[10] Cantrell J.T., Rohde S., Damiani D., Gurnani R., DiSandro L., Anton J., Young A., Jerez A., Steinbach D., Kroese C. (2017). Experimental characterization of the mechanical properties of 3D printed ABS and polycarbonate parts. Rapid Prototyp. J..

[11] Novakova-Marcincinova L., Kuric I. (2012). Basic and Advanced Materials for Fused Deposition Modeling Rapid Prototyping Technology. Manuf. Ind. Eng..

[12] Nordin N.A.B., Johar M.A., Ibrahim M.H.I., Marwah O.M.F. (2017). Advances in high temperature materials for additive manufacturing. IOP Conf. Ser. Mater. Sci. Eng..

[13] Kishore V., Chen X., Ajinjeru C., Hassen A., Lindahl J. (2016). Additive Manufacturing of High. Performance Semicrystalline Thermoplastics and Their Composites.

[14] Fijul Kabir S.M., Mathur K., Seyam A.-F.M. (2020). A critical review on 3D printed continuous fiber-reinforced composites: History, mechanism, materials and properties. J. Comp. Struct..

[15] Skrzypczak N.G., Tanikella N.G., Pearce J.M. (2020). Open source high-temperature RepRap for 3-D printing heat-sterilizable PPE and other applications. HardwareX.

[16] Hunt E.J., Zhang C., Anzalone N., Pearce J.M. (2015). Polymer recycling codes for distributed manufacturing with 3-D printers. Resour. Conserv. Recycl..

[17] Pakkanen J., Manfredi D., Minetola P., Iuliano L. About the Use of Recycled or Biodegradable Filaments for Sustainability of 3D Printing. Proceedings of the International Conference on Sustainable Design Manufacturing.

[18] Tian X., Liu T., Wang Q., Dilmurat A., Li D., Ziegmann G. (2017). Recycling and remanufacturing of 3D printed continuous carbon fiber reinforced PLA composites. J. Clean. Prod..

[19] Zhao P., Rao C., Gu F., Sharmin N., Fu J. (2018). Close-looped recycling of polylactic acid used in 3D printing: An experimental investigation and life cycle assessment. J. Clean. Prod..

[20] Dawoud M., Taha I., Ebeid S.J. (2016). Mechanical behaviour of ABS: An experimental study using FDM and injection moulding techniques. J. Manuf. Process..

[21] Wang X., Zhao L., Fuh J.Y.H., Lee H.P. (2019). Effect of Porosity on Mechanical Properties of 3D Printed Polymers: Experiments and Micromechanical Modeling Based on X-ray Computed Tomography Analysis. Polymers.

[22] Butt J., Bhaskar R. (2020). Investigating the Effects of Annealing on the Mechanical Properties of FFF-Printed Thermoplastics. J. Manuf. Mater. Process..

[23] Rangisetty S., Peel L.D. The Effect of Infill Patterns and Annealing on Mechanical Properties of Additively Manufactured Thermoplastic Composites. Proceedings of the ASME 2017 Conference on Smart Materials, Adaptive Structures and Intelligent Systems.

[24] Slavković V., Grujović N., Dišić A., Radovanović A. Influence of Annealing and Printing Directions on Mechanical Properties of PLA Shape Memory Polymer Produced by Fused Deposition Modeling. Proceedings of the 6th International Congress of Serbian Society of Mechanics.

[25] Wijnen B., Sanders P., Pearce J.M. (2018). Improved model and experimental validation of deformation in fused filament fabrication of polylactic acid. Prog. Addit. Manuf..

[26] Morales N.G., Fleck T.J., Rhoads J.F. (2018). The effect of interlayer cooling on the mechanical properties of components printed via fused deposition. Addit. Manuf..

[27] Paszkiewicz S., Szymczyk A., Pawlikowska D., Irska I., Taraghi I., Pilawka R., Gu J., Li X., Tu Y., Piesowicz E. (2017). Synthesis and characterization of poly(ethylene terephthalate-co-1,4-cyclohexanedimethylene terephtlatate)-block-poly(tetramethylene oxide) copolymers. RSC Adv..

[28] Dupaix R.B., Boyce M.C. (2005). Finite strain behavior of poly(ethylene terephthalate) (PET) and poly(ethylene terephtha-late)-glycol (PETG). Polymer.

[29] Turner S.R., Liu Y., Moeller M., Matyjaszewski K. (2012). Copolyester based on PET with different amounts of CHDM. Polymer Science: A Comprehensive Reference.

[30] Mercado-Colmenero J.M., La Rubia M.D., Mata-Garcia E., Rodriguez-Santiago M., Martin-Doñate C. (2020). Experimental and Numerical Analysis for the Mechanical Characterization of PETG Polymers Manufactured with FDM Technology under Pure Uniaxial Compression Stress States for Architectural Applications. Polymers.

[31] Hassan M.H., Omar A.M., Daskalakis E., Hou Y., Huang B., Strashnov I., Grieve B.D., Bártolo P. (2020). The Potential of Polyethylene Terephthalate Glycol as Biomaterial for Bone Tissue Engineering. Polymers.

[32] Bhundari S., Lopez-Anido R.A., Gardner D.J. (2019). Enhancing the interlayer tensile strength of 3D printed short carbon fiber reinforced PETG and PLA composites via annealing. Addit. Manuf..

[33] ASTM D638—14 Standard Test Method for Tensile Properties of Plastics. https://www.astm.org/Standards/D638.

[34] Popescu D., Zapciu A., Amza C., Marinescu R., Baciu F. (2018). FDM process parameters influence over the mechanical properties of polymer specimens: A review. Polym. Test..

[35] Costa S.F., Duarte F.M., Covas J.A. (2015). Thermal conditions affecting heat transfer in FDM/FFE: A contribution towards the numerical modelling of the process. Virtual Phys. Prototyp..

[36] Phan D.D., Swain Z.R., Mackay M.E. (2018). Rheological and heat transfer effects in fused filament fabrication. J. Rheol..

[37] Rodriguez J.F., Thomas J.P., Renaud J.E. (2000). Characterization of the mesostructure of fused-deposition acrylonitrile—Butadiene—styrene materials. Rapid Prototyp. J..

[38] Hart K.R., Dunn R.M., Sietins J.M., Hofmeister Mock C.M., Mackay M.E., Wetzel E.D. (2018). Increased fracture toughness of additively manufactured amorphous thermoplastics via thermal annealing. Polymer.

[39] Amza C.G., Zapciu A. (2019). Embedding Ultra-High-Molecular-Weight Polyethylene Fibers in 3D printed Polylactic Acid (PLA) Parts. Polymers.

[40] 3DBenchy—The Jolly 3D Printing Torture-Test by CreativeTools.se, License to Use CC BY-ND 4.0. https://www.thingiverse.com/thing:763622.

[41] Benwood C., Anstey A., Andrzejewski J., Misra M., Mohanty A.K. (2018). Improving the Impact Strength and Heat Resistance of 3D Printed Models: Structure, Property, and Processing Correlationships during Fused Deposition Modeling (FDM) of Poly(Lactic Acid). ACS Omega.

[42] Gao X., Qi S., Kuang X., Su Y., Li J., Wang D. (2021). Fused filament fabrication of polymer materials: A review of interlayer bond. Addit. Manuf..

